# Differences in Learning and Persistency Characterizing Behavior in Chronic Pain for the Iowa Gambling Task: Web-Based Laboratory-in-the-Field Study

**DOI:** 10.2196/26307

**Published:** 2022-04-06

**Authors:** Lili Zhang, Himanshu Vashisht, Alekhya Nethra, Brian Slattery, Tomas Ward

**Affiliations:** 1 School of Computing Dublin City University Dublin Ireland; 2 Insight Science Foundation Ireland Research Centre for Data Analytics Dublin City University Dublin Ireland; 3 School of Psychology Dublin City University Dublin Ireland

**Keywords:** chronic pain, decision-making, computational modeling, Iowa Gambling Task, lab-in-the-field experiment

## Abstract

**Background:**

Chronic pain is a significant worldwide health problem. It has been reported that people with chronic pain experience decision-making impairments, but these findings have been based on conventional laboratory experiments to date. In such experiments, researchers have extensive control of conditions and can more precisely eliminate potential confounds. In contrast, there is much less known regarding how chronic pain affects decision-making captured via laboratory-in-the-field experiments. Although such settings can introduce more experimental uncertainty, collecting data in more ecologically valid contexts can better characterize the real-world impact of chronic pain.

**Objective:**

We aim to quantify decision-making differences between individuals with chronic pain and healthy controls in a laboratory-in-the-field environment by taking advantage of internet technologies and social media.

**Methods:**

A cross-sectional design with independent groups was used. A convenience sample of 45 participants was recruited through social media: 20 (44%) participants who self-reported living with chronic pain, and 25 (56%) people with no pain or who were living with pain for <6 months acting as controls. All participants completed a self-report questionnaire assessing their pain experiences and a neuropsychological task measuring their decision-making (ie, the Iowa Gambling Task) in their web browser at a time and location of their choice without supervision.

**Results:**

Standard behavioral analysis revealed no differences in learning strategies between the 2 groups, although qualitative differences could be observed in the learning curves. However, computational modeling revealed that individuals with chronic pain were quicker to update their behavior than healthy controls, which reflected their increased learning rate (95% highest–posterior-density interval [HDI] 0.66-0.99) when fitted to the Values-Plus-Perseverance model. This result was further validated and extended on the Outcome-Representation Learning model as higher differences (95% HDI 0.16-0.47) between the reward and punishment learning rates were observed when fitted to this model, indicating that individuals with chronic pain were more sensitive to rewards. It was also found that they were less persistent in their choices during the Iowa Gambling Task compared with controls, a fact reflected by their decreased outcome perseverance (95% HDI −4.38 to −0.21) when fitted using the Outcome-Representation Learning model. Moreover, correlation analysis revealed that the estimated parameters had predictive value for the self-reported pain experiences, suggesting that the altered cognitive parameters could be potential candidates for inclusion in chronic pain assessments.

**Conclusions:**

We found that individuals with chronic pain were more driven by rewards and less consistent when making decisions in our laboratory-in-the-field experiment. In this case study, it was demonstrated that, compared with standard statistical summaries of behavioral performance, computational approaches offered superior ability to resolve, understand, and explain the differences in decision-making behavior in the context of chronic pain outside the laboratory.

## Introduction

### Background

Chronic pain is defined as pain persisting or reoccurring for more than 3 to 6 months [1] and has been recognized as one of the most significant health issues of the 21st century [2,3]. Approximately 100 million adults in the United States experience chronic pain, resulting in an annual cost of US $560-635 billion in medical treatment and lost productivity [4]. Worldwide, it is estimated that approximately 20% of the population lives with chronic pain [5,6]. As a result, there is significant ongoing research into understanding chronic pain and supporting people who live with this condition. A key area of research is the impact of chronic pain on cognitive or neuropsychological abilities. It has been reported that at least 20% of clinical patients with chronic pain, including those without a history of mental disorders, complain of cognitive impairments that cause significant difficulties in their social life and daily functioning [7]. In other studies, researchers have found that cognitive deficits occur across a range of pain conditions, including fibromyalgia [8], migraine [9], chronic back pain [10], and chronic neuropathic pain [11].

Although pain is an attention-demanding sensory process, cognitive alterations cannot be simply attributed to the extra attentional demand from ongoing pain [10]. Functional magnetic resonance imaging has revealed decreased gray matter density in the medial prefrontal cortex (mPFC) area [12-14] and less brain activation in cortical structures during response inhibition in patients with chronic back pain [15]. These findings are important as the mPFC also plays a critical role in other cognitive functions such as decision-making [16], executive control [17], learning [18], and memory [19]. The latest research findings have confirmed that reduced glutamate in the mPFC significantly impairs emotional and cognitive processing in people with chronic pain [20]. This suggests that chronic pain may have a negative impact on the mPFC and related neural structures and could be considered a cognitive state that may be competing with other cognitive abilities, especially those involving the mPFC such as decision-making, which is one of the cognitive domains in which individuals with chronic pain are commonly impaired.

The Iowa Gambling Task (IGT) developed by Bechara et al [21] is one of the most widely used neuropsychological paradigms for simulating complex and experience-based decision-making. Participants in this task are required to choose cards from one of 4 decks, two of which (decks A and B) are good decks and the remaining 2 (decks C and D) being bad decks. The bad decks yield negative long-term outcomes, whereas the good decks yield positive long-term outcomes. It has been successfully used to distinguish various clinical populations from healthy populations, such as patients with lesions in the ventromedial prefrontal cortex [22,23], obsessive-compulsive disorder [24], and even chronic cannabis use [25-27]. These earlier applications of the IGT found that healthy controls could learn to choose more frequently from good decks than from bad ones, whereas clinical populations tended to more regularly choose from bad decks throughout the task. With relevance to this discussion, the IGT has also been applied to investigate abnormalities in decision-making among people living with various chronic pain conditions, yielding significant findings. After extracting behavioral responses to the IGT, Apkarian et al [12] found that patients with chronic pain more frequently chose cards from bad decks, were less persistent, and exhibited a negative correlation between gambling performance outcome and reported intensity of chronic pain. The participants in the study by Verdejo-García et al [28] were required to complete both the original IGT, where reward was immediate and punishment was delayed, and an IGT variant where punishment was immediate and reward was delayed. The authors summarized their behavioral choices and found that women with fibromyalgia had significantly lower scores on the third block, which was referred to as the hunch period of the task, on the original IGT, whereas intact performance on the IGT variant suggested that these patients were hypersensitive to rewards. Similar results were obtained in the study by Tamburin et al [10], where people with chronic back pain won significantly less money relative to healthy controls, and their IGT scores did not change significantly throughout the task.

### Objective

It is worth noting that all the relevant studies to date have been conducted in a laboratory setting. In these settings, the participants were under tight experimental control. No study to date has investigated decision-making tasks such as the IGT in the context of chronic pain in more natural environments where the experimenter has much less control. A laboratory-in-the-field approach is adopted in this study and, although such a setting can introduce experimental *noise* and potential confounds that may bias the results, it is closer to observing more representative behavior for this population [29]. In addition, carrying out web-based behavioral experiments is the only entirely risk-free method currently available under the typical movement restrictions imposed by the threat of COVID-19. Thus, in this study, we are interested in investigating the differences in characterizations of decision-making between individuals with chronic pain and normal controls in their everyday living environment. We are also interested in the analysis approaches that are best able to extract behavioral signals in *noisy* experimental environments. In terms of the experimental task, the participants were required to complete the pain assessments and the IGT on their web browser in an environment where they carried out the task at a location of their choice, at a time of their choice, and without supervision. To the best of our knowledge, this is among the first research studies investigating chronic pain through internet-based technology combining decision-making tasks and self-reports.

Given the higher variability of data when collection takes place outside a laboratory setting, conventional behavioral data statistics may not be sufficient to reveal signals in the noisier data collected. In previous studies, this conventional analysis has been based on the measurement of the proportion of choices from the good decks relative to the bad decks. Furthermore, these behavioral summaries are agnostic to the underlying cognitive mechanisms that drive the behavioral performance on the IGT, thus limiting their interpretability. Therefore, in this study, we apply computational modeling analysis as a complementary approach to explicitly decompose behavioral performance on the IGT into cognitive parameters. It has been documented that estimated parameters from such models are able to reveal group differences in cognitive processes despite the absence of group differences in conventional IGT measurements [30]. The extracted parameters can then be used to understand the source of the decision-making alterations. We hypothesize that the noisier experimental environment might produce data that reveal little difference between the experimental groups when considered through conventional analysis, whereas the *filtering* enabled by the computational modeling might reveal significant differences in some cognitively interpretable parameters. Although computational modeling analyses have been successfully applied to capture the complex interplay of cognitive processes for people with a variety of other health issues [31,32], we have only found 1 reference in which a simple heuristic model was designed to differentiate the behavioral performance of individuals with chronic pain and healthy controls on the IGT [33]; thus, the changes in latent cognitive parameters that drive the impaired performance of people living with chronic pain remain unexplored until now. Given that more competitive cognitive models and more advanced parameter estimation methods have been developed to more precisely characterize the underlying cognitive mechanisms, we hypothesize that computational modeling analysis is more effective in capturing the differences in decision-making from the data set collected in a laboratory-in-the-field environment.

## Methods

### Recruitment

The participants in this study were recruited through social media and local pain advocacy groups. A total of 64 people, including 28 (44%) symptomatic participants who had lived with chronic pain for months and 36 (56%) healthy controls who had never lived with chronic pain or had experienced pain for <6 months, were interested in participating in the experiments. They were directed to a webpage containing the plain language statement of the experiment. After providing informed consent, they were linked to a questionnaire and the IGT through their computer or mobile phone. A total of 8 symptomatic participants and 11 healthy controls were excluded from the study as they failed to complete the IGT. Thus, a convenience sample of 45 participants (45/64, 70% of the total) was recruited finally, consisting of 20 (44%) symptomatic participants (14/20, 70% women; mean age 40, SD 12 years) and 25 (56%) healthy controls (12/25, 48% women; mean age 38, SD 12 years). The groups did not significantly differ in age (*P*=.43) or proportion of women (*P*=.07).

### Ethics Approval

This study was approved by the local ethics committee of the School of Computing, Dublin City University (DCUREC/CA/2019/1).

### Assessment of Pain Experience

**Figure 1:**
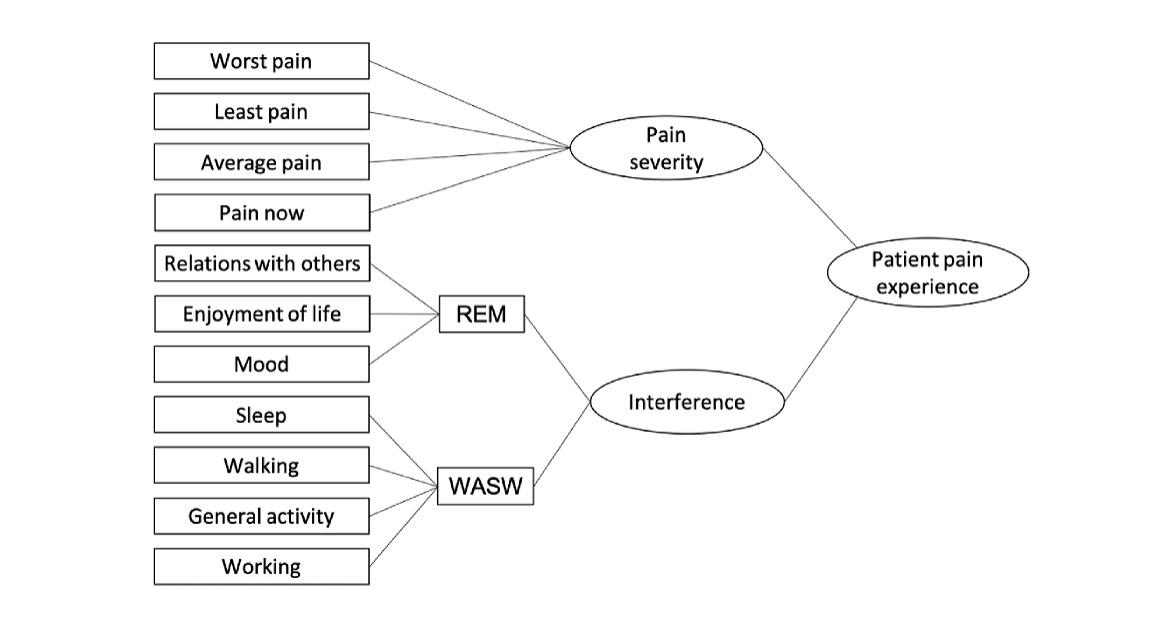
A graphical representation of the conceptual framework of the measurement. REM: relations with others, enjoyment of life, and mood subdimension of the Brief Pain Inventory–Short Form; WASW: walking, general activity, sleep, and work subdimension of the Brief Pain Inventory–Short Form.

The Brief Pain Inventory–Short Form (BPI-SF) is a validated, 9-item self-administered questionnaire used to evaluate the severity of the patient’s pain and its impact on the patient’s daily functioning. The patient is asked to rate their worst, least, average, and current pain intensity; list current treatments and their perceived effectiveness; and rate the degree to which pain interferes with general activity, mood, walking ability, normal work, relations with other persons, sleep, and enjoyment of life on a 10-point scale. The pain interference is then divided into affective subdimensions (ie, relations with others, enjoyment of life, and mood [REM]) and activity subdimensions (ie, walking, general activity, sleep, and work [WASW]). The BPI-SF has been used with a variety of populations and has been shown to be a valid and reliable measure with adequate internal reliability across these studies (eg, *α*=.86-.96) [34]. A graphical representation of the conceptual framework of the measurement is shown in Figure 1.

### The IGT Paradigm

Participants in the IGT are initially given €2000 (US $2216.10) in virtual money and presented with 4 decks of cards labeled A, B, C, and D. Each card in these decks can generate gains and sometimes cause losses. Participants have to choose 1 card from these 4 decks consecutively until the task shuts off automatically after 100 trials. In each trial, feedback on the rewards and losses of their choice and the running tally over all trials so far are given to the participants, but no information is given regarding how many trials they will play and how many trials they have completed during the task. Participants are instructed that they can choose cards from any deck and switch decks at any time. They are also told to make as much money as possible, thus minimizing losses.

**Figure 2:**
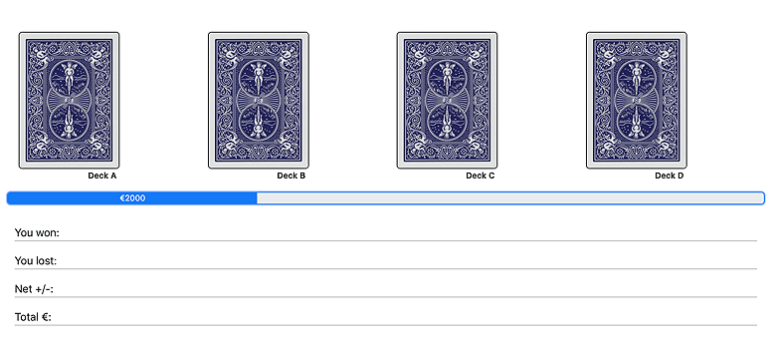
Screenshot of the web-based Iowa Gambling Task.

Table 1 shows the payoffs of the 4 decks. As can be seen in the table, decks A and B are 2 bad decks that generate high immediate, constant rewards but even higher unpredictable, occasional losses. Thus, the long-term net outcome associated with decks A and B is negative. In contrast, decks C and D are 2 good decks that generate low immediate, constant rewards but even lower unpredictable, occasional losses. Thus, the long-term net outcome associated with decks C and D is positive. In addition to the payoff magnitudes, the 4 decks also differ in the frequency of losses (ie, decks A and C are associated with a higher frequency of losses, whereas decks B and D are associated with a lower frequency of losses). The key to obtaining a higher long-term net outcome in this task is to explore all the decks in the initial stage and then exploit the 2 good decks (see Figure 2 for the screenshot of the web-based IGT that we implemented).

**Table 1:** Summary of the payoff of the Iowa Gambling Task.

	Deck A (bad deck with frequent losses)	Deck B (bad deck with infrequent losses)	Deck C (good deck with frequent losses)	Deck D (good deck with infrequent losses)
Reward/trial (€)	100	100	50	50
Number of loss trials/10 trials	5	1	5	1
Loss/10 trials (€)	−1250	−1250	−250	−250
Net outcome/10 trials (€)	−250	−250	250	250

### Standard Behavioral Data Analysis

To compare the behavioral differences between the 2 groups in the IGT, 3 parameters were measured. First, the total amount of gain at the end of the task was calculated for each participant to measure their overall performance on the task. An unpaired 2-tailed *t* test was used to determine if the difference between the 2 groups was significant on this measure. Second, to obtain a visual exploration of the group-level deck preferences across trials, we calculated the proportions of good deck selections (decks C and D) and the learning IGT scores (ie, the difference between the number of good deck selections [decks C and D] and the number of bad deck selections [decks A and B]) across the task. Specifically, 5 new variables were created through the division of the 100 trials into 5 blocks of 20 trials each without overlap. The proportion of good deck selections and the difference between the number of good deck selections and the learning score in each block were calculated. In this way, 5 proportions and 5 learning scores, 1 for each block, were obtained for each participant. The comparison between the 5 learning scores is regarded as an index of learning. A learning score increasing from the first block to the last block suggests that a participant is developing a preference for good decks and an effective selection strategy. Given the repeated learning scores of the 2 groups over the 5 blocks of trials, a block-by-group Bayesian repeated-measure analysis of variance was performed (within-participant factor: block 1-5; between-participant factor: group healthy vs chronic pain group) to reveal whether the 2 groups differed in learning curves.

### Computational Modeling Analysis

#### Overview

Poor performance on the IGT can be due to a variety of altered underlying neurocognitive processes such as poor learning, memory, hypersensitivity to rewards and losses, or less response consistency. To more precisely identify the psychological processes that drive participants’ behavioral performances on the IGT, multiple cognitive models have been proposed, such as the Expectancy–Valence Learning model (which is also the first proposed cognitive model for the IGT) [35], the Prospect Valence Learning model with Delta (PVL-Delta) [36], the PVL model with decay (PVL-Decay) [26], and the Values-Plus-Perseverance (VPP) model [26] (these 3 models are derived from the original Expectancy–Valence Learning model but show better performance), as well as the recently proposed Outcome-Representation Learning (ORL) model [37] (see Textbox 1 for the parameter specifications of the 4 IGT models). We fitted the 4 models using the hBayesDM package in R (R Foundation for Statistical Computing) [38].

**Parameter specifications of the 4 Iowa Gambling Task models.**

**Models and their parameters**

- Prospect Valence Learning (PVL)-Delta (four parameters): outcome sensitivity (α), loss aversion (λ), learning rate (A), and response consistency (c)
- PVL-Decay (four parameters): outcome sensitivity (α), loss aversion (λ), decay parameter (A), and response consistency (c)
- Values-Plus-Perseverance (eight parameters): outcome sensitivity (α), loss aversion (λ), learning rate (A), decay parameter (K), gain impact parameter (EP_p_), loss impact parameter (EP_N–_), weight parameter (w), and response consistency (c)
- Outcome-Representation Learning (five parameters): reward learning rate (A_+_), punishment learning rate (A_–_), decay parameter (K), outcome frequency weight (β_F_), and perseverance weight (β_p_)

#### The PVL Models

Both the PVL-Delta and PVL-Decay models consist of a utility function, a learning rule, and an action selection rule. They are identical except that they use different learning rules. The utility function determines the weight given to gains relative to losses. Both PVL variants applied the Prospect utility function [39] that featured diminishing sensitivity to increases in magnitude and different sensitivity to losses versus gains. The utility *u*(*t*) of each net outcome *x*(*t*)—that is, the difference between the amount of rewards and losses—in trial *t* is calculated as follows: 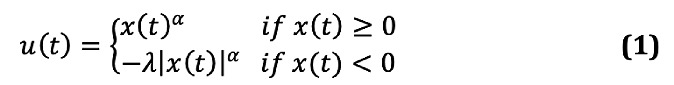 Where *u*(*t*) is the subjective utility of the experienced net outcome *x*(*t*), *α* is the outcome sensitivity parameter (0<*α*<1) that controls the shape of the utility function, and *λ* is the loss aversion parameter (0<*λ*<10) that governs the sensitivity to losses relative to gains. A higher value of *α* suggests that individuals have greater sensitivity to feedback outcomes. A value of *λ*<1 indicates that individuals are more sensitive to gains than to losses, and a value of *λ*>1 indicates that they are more sensitive to losses than to gains.

The learning rule in the PVL models is used to update the expectancies of the decks *E*(*t*) based on the subjective utility value. In the delta rule, a simplified version of the Rascorla-Wagner rule is applied, in which only the expectancy of the chosen selection is updated, whereas the expectancies for other decks remain unchanged: *E_i_*(*t* + 1) = *E_j_*(*t*) + *A ·* (*u*(*t*) – *E_j_*(*t*)) (equation 2), where *A* is the learning rate parameter (0≤*A*≤1) that determines how much weight the decision maker gives to the recent outcomes when updating expectancies. However, in the decay rule, the expectancies of all decks are discounted in each trial except for the chosen deck, which is updated by the current outcome utility: *E_i_* (*t* + 1) = *A* · *E_j_*(*t*) + *δ_j_*(*t*) · *u*(*t*) (equation 3).

Here, *A* is the decay parameter (0≤*A*≤1) that determines how much the past expectancy is discounted, and *δ_j_*(*t*) is a dummy variable that equals 1 when deck *i* is chosen and 0 otherwise.

The action selection rule generates the choice possibilities Pr(*D*(*t* + 1) = *i*) for each deck in the next trial using a softmax function: 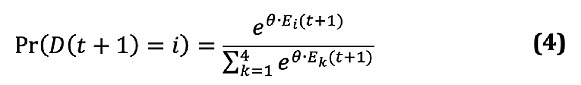 Where *D*(*t*) is the chosen deck on trial *t*, *θ* is assumed to be trial-independent and set to 3*^c^* – 1, and *c* (0≤*c*≤5) is a response consistency parameter. A higher value of *c* indicates that the decision maker has a higher tendency to select choices with higher expected values, which means that they are responding more deterministically.

#### VPP Model

The VPP model adds a perseveration term *P_i_*(*t*) for the chosen deck *i* on trial *t* based on the PVL-Delta model:

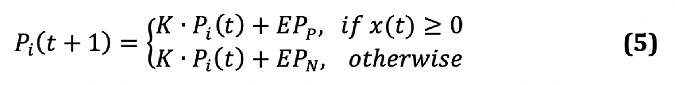



*K* is a decay parameter that determines how much the perseveration value of each deck is discounted in each trial. The tendency to perseverate or switch is incremented each time and updated by a gain impact parameter *EP_p_* (*–Inf* < *EP_p_* < *Inf*) and a loss impact parameter *EP_N_* (*–Inf* < *EP_N_* < *Inf*) based on whether the net outcome in the previous trial was a loss or a gain. Positive values for these parameters indicate stronger tendencies for decision makers to perseverate the deck chosen in the previous trial, whereas negative values indicate switching tendencies.

The expected value and perseveration term are then integrated into a single-value signal: *V_i_*(*t* + 1) = *w* · *E_i_*(*t* + 1) + (1 – *w*) · *P_i_*(*t* + 1) (equation 6), where *w* (0<*w*<1) is a weight parameter that controls the weight given to the expected value in each trial. A greater value of *w* represents a greater weight given to the expected value. The values of *V_i_* (*t* + 1) are then entered into the softmax function to calculate the probabilities of each option being chosen.

#### ORL Model

The recently proposed ORL model assumes that people track the expected value (*EV*(*t*)) and the win frequency (*EF*(*t*)) separately. In addition, for positive and negative net outcomes, the decision makers update the expectancy of the chosen deck *i* with different learning rates: 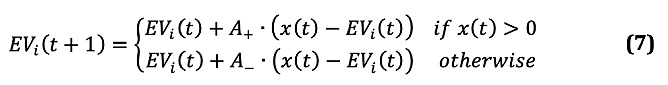 Here, *A*_+_ (0<*A*_+_<1) and *A*_–_ (0<*A*_–_<1) are the reward and punishment learning rates, respectively, used to update the expected value of the chosen deck after rewards and punishment. The updating process for the win frequency (*EF*(*t*)) of the chosen option is as follows: 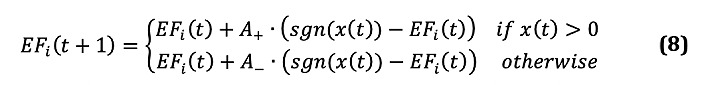 Here, *A*_+_ and *A*_–_ are the same learning rates as those used to update the expected value, and *sgn*(*x*(*t*)) returns 1, 0, or −1 for positive, 0, or negative outcome values on trial *t*, respectively. The expected outcome frequencies for unchosen decks *j’* are also updated in each trial, in which the learning rates are also shared from the expected value learning rule:

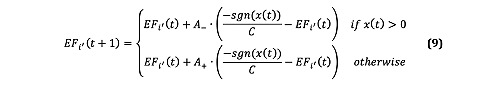



Here, *c* is the number of alternative choices for the chosen deck *j*, which is 3 in the case of the IGT. The ORL model also assumes that the decision makers have tendencies to stay or switch their choices regardless of the outcome in the last trial, and this tendency can be captured by a perseverance weight (*PS_i_*(*t*)): 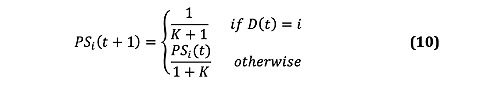 Here, *K* is the decay parameter that controls how quickly the past deck selections are forgotten and is determined by *K* = 3*^K’^* – 1 (equation 11).

*K’* ∈ [0,5], so *K* ∈ [0,242]. A single-value signal for each deck is then produced by integrating the expected value, frequency, and perseverance into a linear function: *V_i_*(*t* + 1) = *EV_i_*(*t* + 1) + *EF_i_*(*t* + 1) · *β_F_* + *PS_i_*(*t* + 1) · *β_p_* (equation 12).

*βF (–Inf < βF < Inf)* and *βp (–Inf < βp < Inf)* are 2-weight parameters that reflect the weight given to the outcome frequency and perseverance, respectively, relative to the expected value of each deck. Finally, the probability of each choice is determined by passing the expected values through the softmax function:

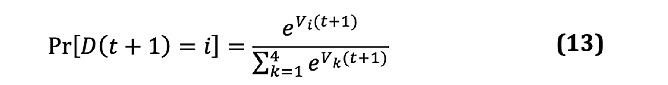



## Results

### Overview

Individuals living with chronic pain (n=20) and healthy controls (n=25) completed the web-based BPI-SF and the original version of the IGT at a location of their choice and at a time of their choice without supervision. The full demographic information is presented in Table 2.

**Table 2:** Demographic information for the healthy controls and people living with chronic pain (N=45).

Characteristic	Healthy controls (n=25),^a^ mean (SD; range)	Participants with chronic pain (n=20),^b^ mean (SD; range)	*P* value^c^
Age (years)	37.2 (12.5; 24-63)	40.2 (11.9; 23-62)	.43
Pain duration (months)	1.2 (1.3; 0-5)	78.6 (76.3; 6-264)	<.001
Pain severity	2.07 (2.5; 0-10)	4.3 (2.3; 0-8)	.004
Interference (REM^d^)	2.0 (2.9; 0-9.3)	4.6 (3.3; 0-9)	.007
Interference (WASW^e^)	1.9 (2.6; 0-8.3)	4.2 (2.5; 0-8.5)	.004

^a^12 females.

^b^14 females.

^c^*P* value for females was .07.

^d^REM: relations with others, enjoyment of life, and mood subdimension of the Brief Pain Inventory–Short Form.

^e^WASW: walking, general activity, sleep, and work subdimension of the Brief Pain Inventory–Short Form.

### Self-report Analysis

**Figure 3:**
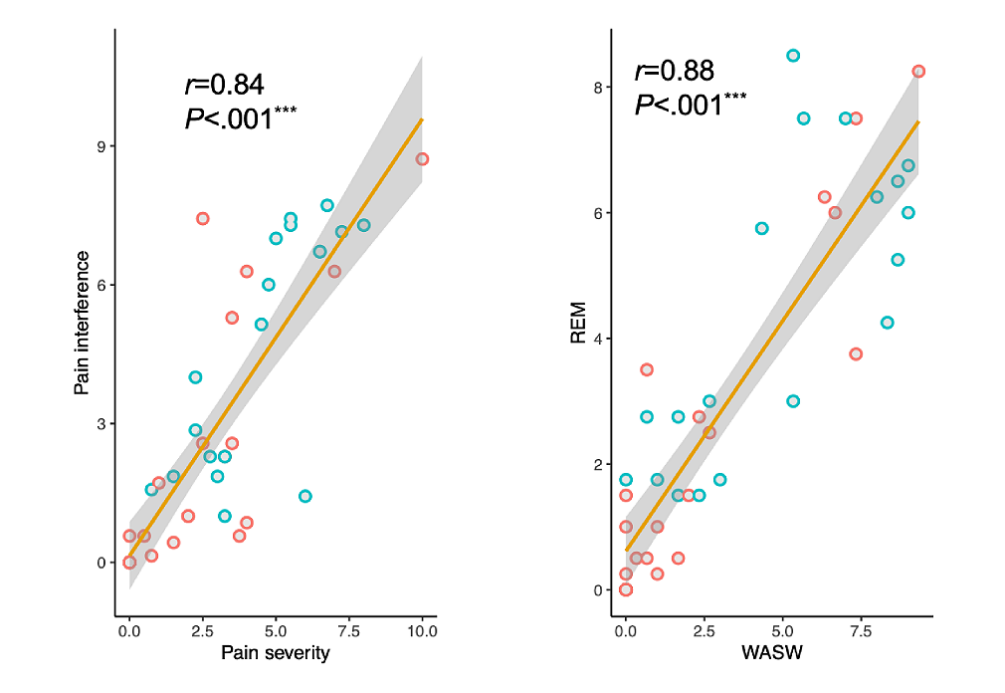
Pain severity plotted against pain interference (left) and activity subdimension plotted against affective subdimension (right). Healthy controls (n=25) are plotted in red, and people living with chronic pain (n=20) are plotted in blue. The *r* values were calculated between the paired pain measures for the whole sample (*P<.5, **P<.01, ***P<.001). REM: relations with others, enjoyment of life, and mood subdimension of the Brief Pain Inventory–Short Form; WASW: walking, general activity, sleep, and work subdimension of the Brief Pain Inventory–Short Form.

As expected, individuals with chronic pain demonstrated higher levels of pain severity (*t*_43_=–3.06; *P*=.004; Cohen *d*=–0.92, 95% CI −3.64 to −0.75), and their daily activities were more influenced by pain (*t*_43_=–3.05; *P*=.004; *d*=–0.92, 95% CI −4.1 to −0.83) in comparison with healthy controls. Moreover, individuals living with chronic pain reported higher levels of subdimensional interference in REM (*t*_43_=–2.86; *P*=.007; *d*=–0.86, 95% CI −4.50 to −0.78) and WASW (*t*_43_=–3.02; *P*=.004; *d*=–0.91, 95% CI −3.84 to −0.77). As expected, pain severity was strongly correlated with pain interference (*r*_40_=0.78, 95% CI 0.63-0.88; log*BF*_10_=7.18; *P*<.001). The 2 subdimensional interferences were positively correlated (*r*_40_=0.87, 95% CI 0.76-0.93; log*BF*_10_=10.8; *P*<.001) as well (Figure 3).

### Standard Behavioral Data Analysis

**Figure 4:**
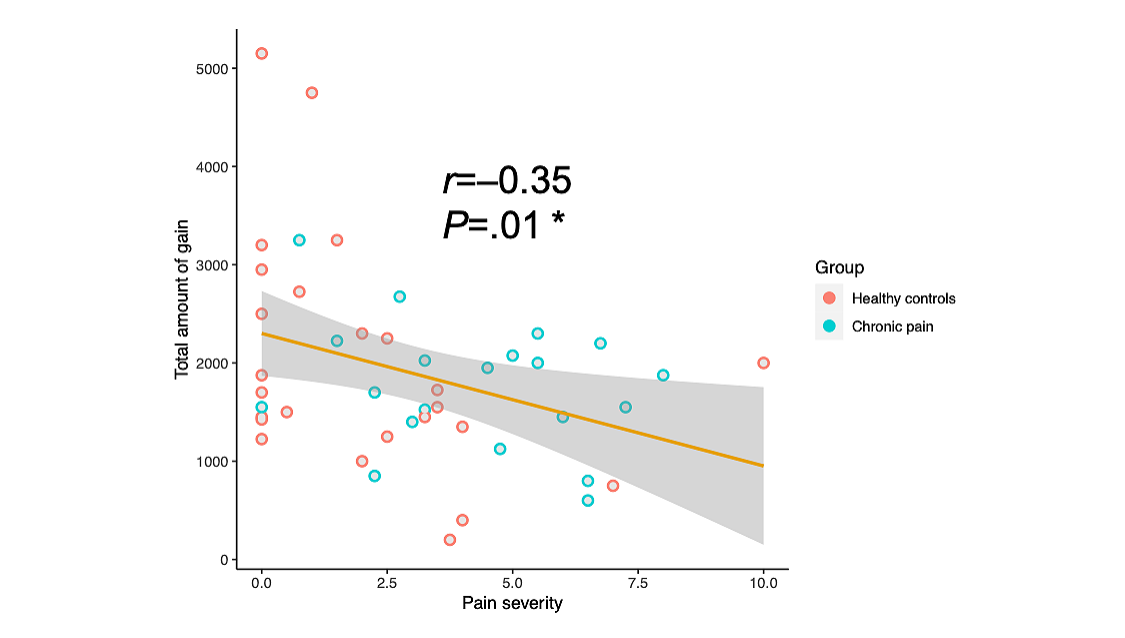
Pain severity plotted against total amount of gain by the end of the task. Healthy controls (n=25) are plotted in red, and people living with chronic pain (n=20) are plotted in blue. The *r* value was calculated between the pain measure and the task performance measure for the whole sample (*P<.5, **P<.01, ***P<.001).

**Figure 5:**
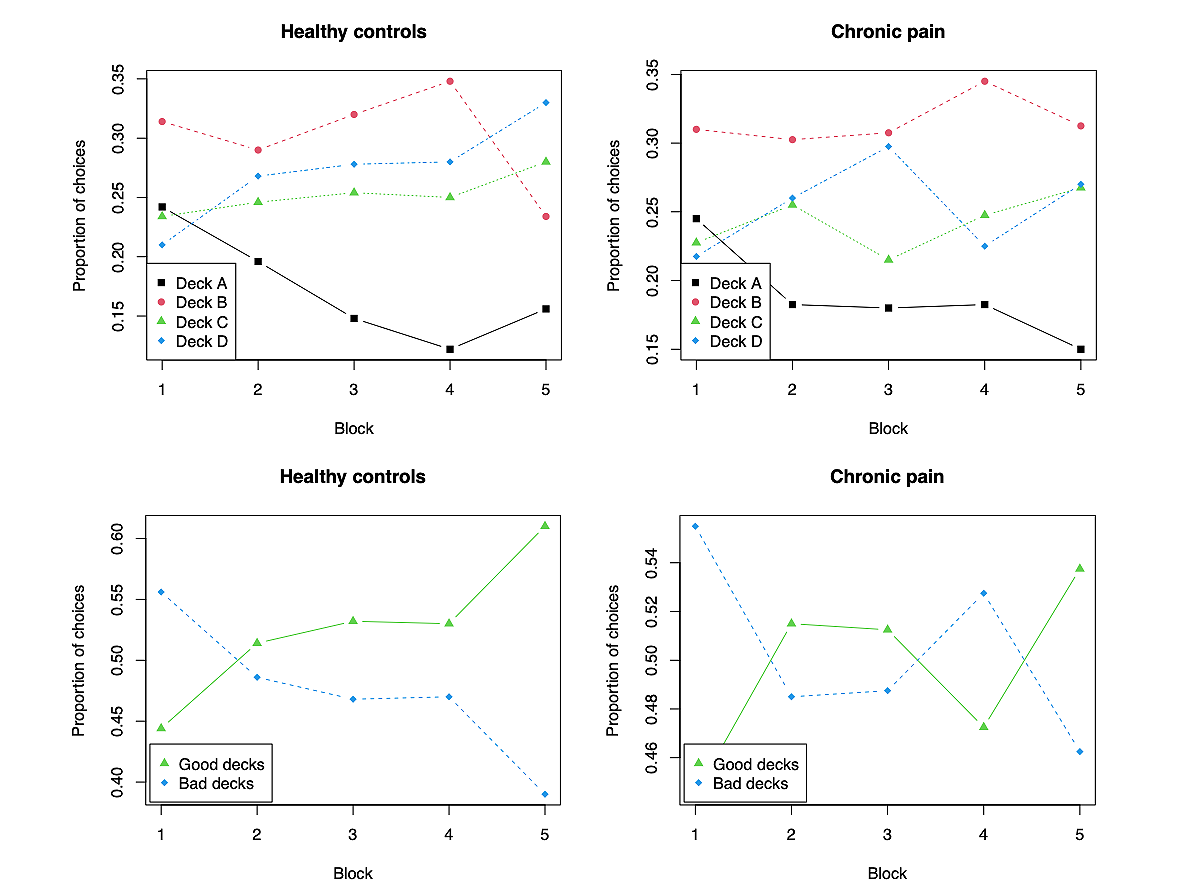
Mean proportion of choices from each deck within 5 blocks of both groups of decision makers (top 2 graphs) and mean proportion of choices from good decks and bad decks of both groups of decision makers (last 2 graphs). Each block contains 20 trials.

The total amount of gain at the end of the IGT did not significantly differ between individuals with chronic pain (€; mean 1997, SD 1187) and healthy controls (mean 1756, SD 645; *t*_43_=0.81; *P*=.42). However, pain severity was significantly correlated with total gain (*r*_43_=–0.39, 95% CI −0.62 to −0.11; *P*=.008; Figure 4). Figure 5 shows the proportion of choices from each deck as a function of the 5 blocks for the healthy and chronic pain groups separately and the proportion of choices from the good and bad decks. The choice pattern of the chronic pain group was qualitatively different (visual inspection of plots) from that of the healthy controls, although both groups demonstrated a clear avoidance of bad deck A. People with chronic pain showed an obvious preference for disadvantageous deck B. In contrast, healthy controls consistently favored deck D as the task progressed. Both decks B and D, which featured low-frequency losses, were generally chosen more often than decks A and C, which featured high-frequency losses. Decision makers both healthy and with chronic pain selected more cards from good decks than from bad decks at the beginning of the task. After learning whether each deck was good or bad in the second block, the healthy controls continued to select more from good decks than from bad decks. However, the choices of decision makers with chronic pain seemed to fluctuate more across the advantageous and disadvantageous decks throughout the task. The final proportion of good deck selection of healthy decision makers was higher than that of decision makers with chronic pain.

**Figure 6:**
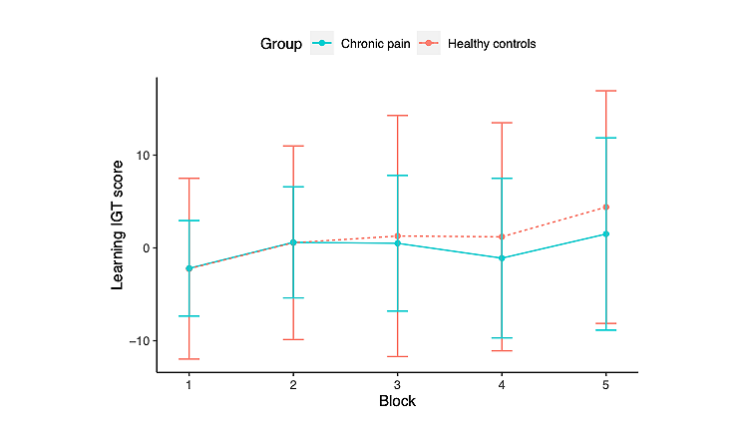
The learning Iowa Gambling Task (IGT) scores across the 5 different blocks of the IGT in healthy controls and people living with chronic pain.

Figure 6 shows the learning scores across the 5 blocks of the IGT. A learning process was apparent in the healthy control group, in which the learning score progressively improved across the 5 blocks. Although the learning scores of individuals with chronic pain also showed an increasing trend, there was a clear dip in block 4. It is worth pointing out that the choice variances of the 2 groups in our study, as shown in Figure 6, were both higher than the variances reported in previous studies that administered the IGT in a laboratory setting [10,40]. This is evidence perhaps of the additional noise introduced by the unsupervised experimental setting. To quantify the group differences, we applied the Bayesian repeated-measure analysis of variance test in the form of a 5 (block) × 2 (health status) to the learning scores (Table 3). To our surprise, the results showed that neither the block nor the group factor had a significant impact on deck selection because the evidence in favor of the null hypothesis was 3.33:1 in favor of the alternative hypothesis that assumes an effect of group and 1.45:1 in favor of the alternative hypothesis that assumes an effect of block. This suggests that people living with chronic pain and healthy controls did not show significant deck preferences in the IGT, and neither group developed a strong learning curve during the task. We will discuss the possible implications of this observation in the *Discussion* section.

**Table 3:** Output of the Bayesian repeated-measure analysis of variance conducted in JASP.

Model	*BF*_01_
Null model	*1.000* ^a^
Group	3.33
Block	1.45
Block + group	4.65
Block + group + block × group	90.91

^a^1: no evidence; 1-3: anecdotal evidence for H0; 3-10: moderate evidence for H1; 10-30: strong evidence for H1; 30-100: very strong evidence for H1.

### Computational Modeling Analysis for the IGT

Although the behavioral data statistics suggest that decision makers both healthy and with chronic pain did not show significantly different deck preferences in the IGT, there might still be group differences in the cognitive processes underlying the choices. To investigate this possibility, we decomposed the IGT performance of the 2 groups using the cognitive modeling analysis introduced earlier.

We first checked which model provided the best short-term prediction performance as measured by the one-step-ahead leave-one-out information criterion (LOOIC). The smaller a model’s LOOIC score is, the better its model fit is. As shown in Table 4, the VPP model demonstrated the best overall model fit relative to other models followed by the ORL model. The ORL model ranked best in the chronic pain group.

**Table 4:** Models and prior fits.

Model	Pain LOOIC^a^	Healthy LOOIC	Sum LOOIC
IGT^b^ ORL^c^	*4593^d^*	4600	9194
IGT VPP^e^	4623	*4544*	*9168*
IGT PVL^f^-Decay	4756	4867	9624
IGT PVL-Delta	5054	5622	10,676

^a^LOOIC: leave-one-out information criterion.

^b^IGT: Iowa Gambling Task.

^c^ORL: Outcome-Representation Learning.

^d^The smaller the value, the better the model fits the data.

^e^VPP: Values-Plus-Perseverance.

^f^PVL: Prospect Valence Learning.

**Figure 7:**
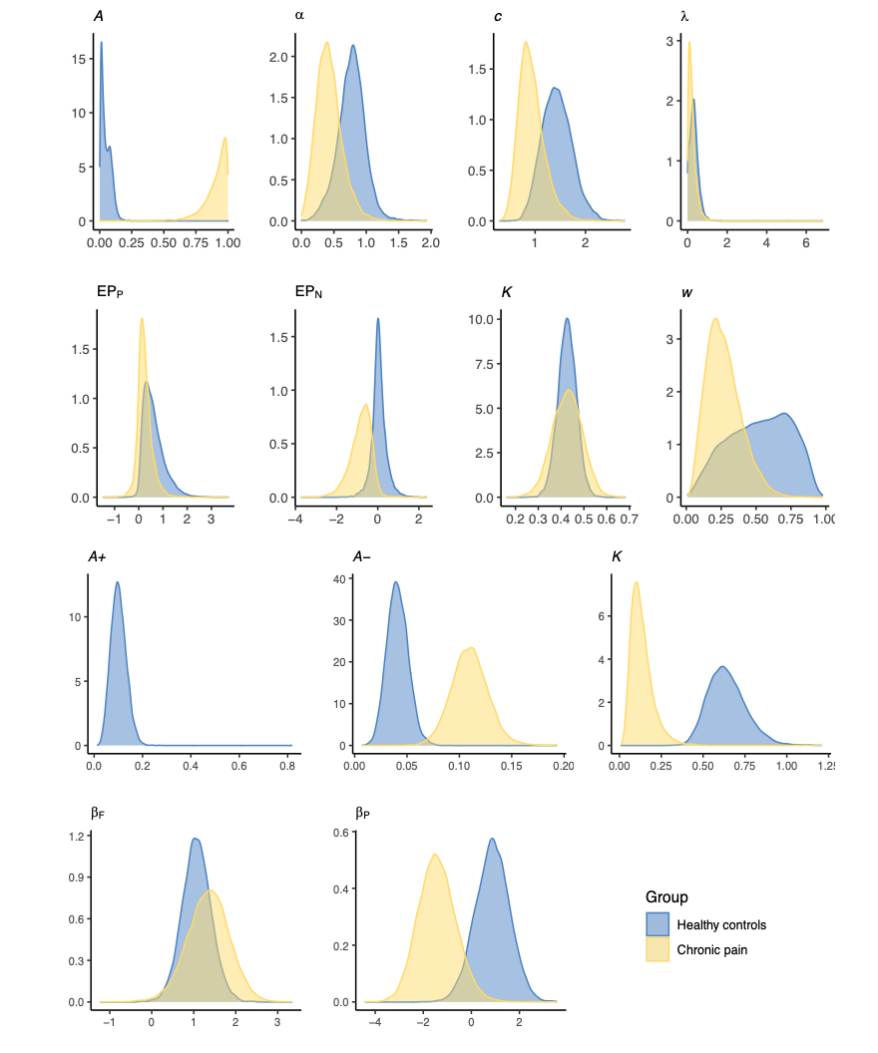
Group-level Values-Plus-Perseverance (top panel) and Outcome-Representation Learning (bottom panel) parameters across healthy controls and people living with chronic pain.

**Figure 8:**
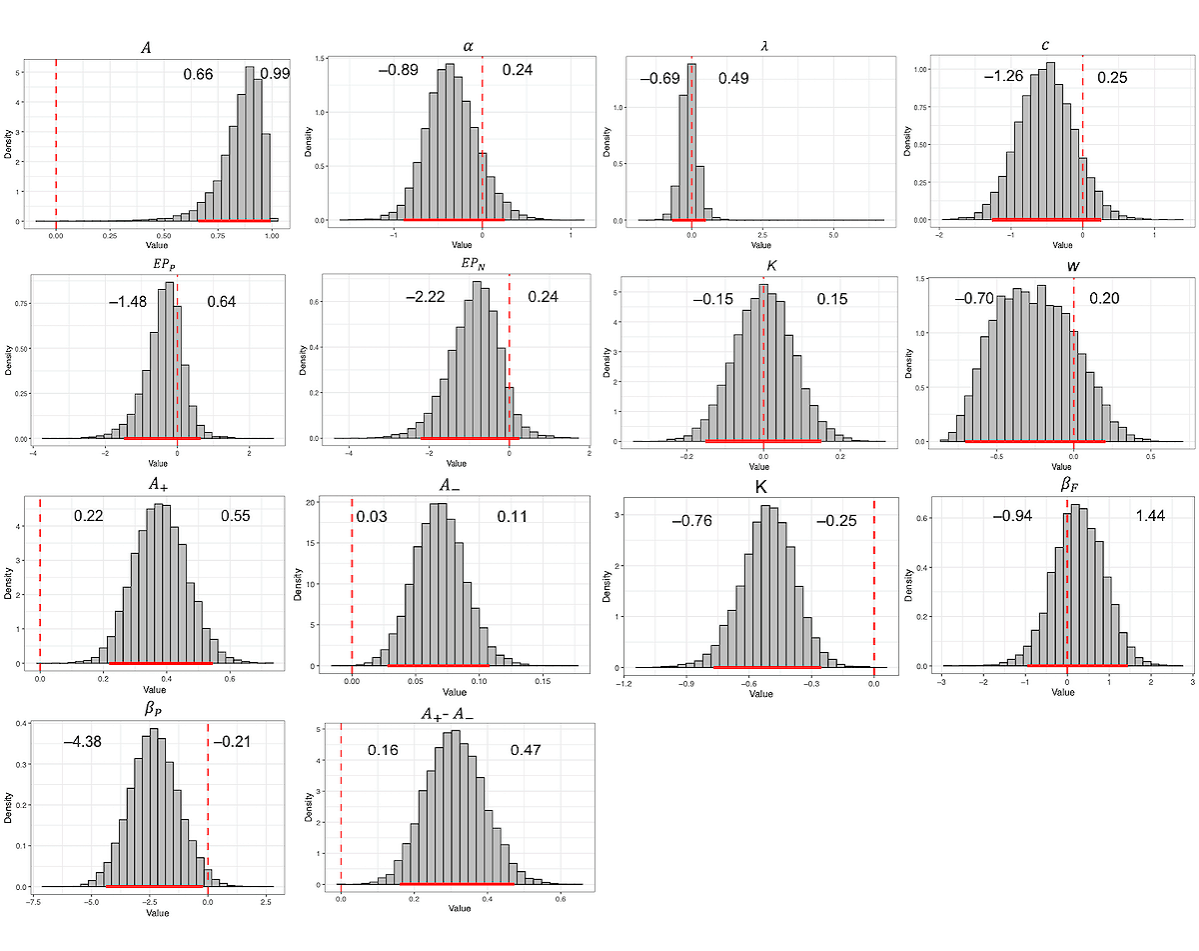
Differences in group-level Values-Plus-Perseverance (VPP) and Outcome-Representation Learning (ORL) parameter distribution between healthy and symptomatic groups. Solid red lines covered the 95% highest–posterior-density interval (HDI), and dashed red lines marked the 0 point. Values on the left and right sides of each graph are the lower and upper bounds of the 95% HDI of the comparison between the symptomatic and healthy control groups. If 0 point is included in the HDI, we consider there to be a nonsignificant difference between the groups.

Next, we used the best-fitting models (VPP and ORL) to compare the 2 groups. Figure 7 shows the posterior distributions of the group-level mean parameters of the VPP and ORL models fitted with two priors (one for each group) separately for healthy decision makers and decision makers with chronic pain. The extracted parameters for the VPP model demonstrated significantly elevated learning rates in individuals with chronic pain relative to normal controls (95% highest–posterior-density interval [HDI] 0.66-0.99). The chronic pain group also showed strong evidence of increased reward learning rate (95% HDI 0.22-0.55), punishment learning rate (95% HDI 0.03-0.11) and difference between the reward and punishment learning rate (95% HDI 0.16-0.47), and decreased decay rate (95% HDI −0.76 to −0.25) and outcome perseverance (95% HDI −4.38 to −0.21) than healthy controls when fitting to the ORL model (the 95% HDI for the comparison across groups did not overlap zero; Figure 8).

**Figure 9:**
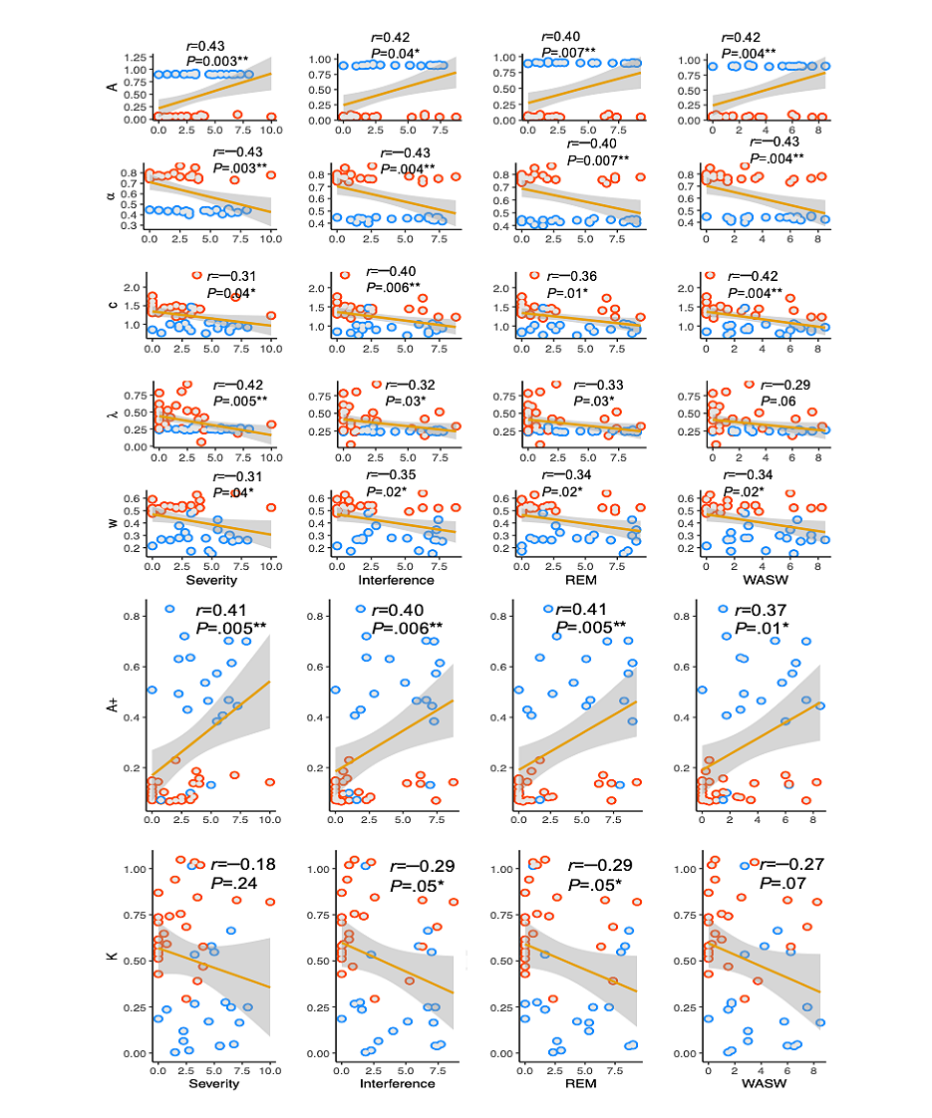
Estimated parameters of the Values-Plus-Perseverance model and Outcome-Representation Learning model plotted against 4 pain measures. Healthy controls are plotted in red, and patients with chronic pain are plotted in blue. The *r* values were calculated between the model parameters and pain measures for the whole sample (*P<.05, **P<.01, ***P<.001). REM: relations with others, enjoyment of life, and mood subdimension of the Brief Pain Inventory–Short Form; WASW: walking, general activity, sleep, and work subdimension of the Brief Pain Inventory–Short Form.

Extracting each individual’s posterior mean estimated parameters for the VPP model, shrinkage effects [41] were observed in the individual estimations of the learning rate and outcome sensitivity parameter in Figure 9, in which the individual estimations of these 2 parameters were shrunk toward the population mean. Significant evidence of positive correlations was identified between learning rate (*A*) and pain severity (*r*_43_=0.43, 95% CI 0.15-0.64; log*BF*_10_=2.48; *P*=.003), average pain interference (*r*_43_=0.42, 95% CI 0.15-0.64; log*BF*_10_=2.37; *P*=.04), and the 2 subdimensional interferences. However, a negative correlation was observed between the 4 pain measures and the outcome sensitivity (*α*), response consistency (*c*), loss aversion (*λ*), and weight parameter (*w*) except for WASW and loss aversion. We did not find significant correlations between the other 3 parameters in the VPP model and the pain measures.

Extracting individual estimations for the ORL model provided evidence for the existence of positive correlations between pain severity and reward learning rate (*r*_43_=0.41, 95% CI 0.12-0.62; log*BF*_10_=2.18; *P*=.005). Similar correlations were obtained between pain interference (including 2 subdimensional interferences) and this model parameter. However, a negative correlation was observed between pain interference and the decay rate parameter (*r*_43_=–0.29, 95% CI −0.54 to −0.00; log*BF*_10_=0.18; *P*=.05), but this correlation with the decay rate parameter did not apply to pain severity. A similar correlation was only identified between REM and the decay rate parameter (*r*_43_=–0.29, 95% CI −0.54 to −0.00; log*BF*_10_=0.18; *P*=.05), but no supported correlation for WASW (*r*_43_=–0.27, 95% CI −0.53 to −0.02; log*BF*_10_=–0.04; *P*=.07) was identified.

## Discussion

### Principal Findings

In this study, we explored the differences in decision-making between individuals living with chronic pain and healthy controls in a laboratory-in-the-field environment by collecting their behavioral responses to the web-based IGT. The main finding of our study is that people with chronic pain did not show significant differences in decision-making in the IGT based on standard behavioral statistics. However, further computational modeling analysis revealed that people with chronic pain had an elevated learning rate for rewards and punishments when fitting to the VPP model. Further results were obtained when fitting the data to the ORL model, where individuals with chronic pain were more dominated by rewards. Meanwhile, the symptomatic group demonstrated a decreased decay rate and perseverance weight when fitting to the ORL model. We also explored the connection between the self-reported pain experiences, standard inferential statistics, and cognitive parameters. The main finding is that the total amount of gain at the end of the task was negatively correlated with the degree of pain severity. Moreover, several cognitive parameters could also predict pain severity and pain interference assessed by the self-reported pain experiences.

Using standard inferential statistics, the total amount of gain obtained by the end of the task was not significantly lower in people living with chronic pain than in healthy controls, indicating that there was no difference between the 2 groups in terms of the overall performance on the IGT. However, the total amount of gain was negatively correlated with the pain severity measure, suggesting that higher pain severity could be a factor that impairs performance on the IGT. Although people with chronic pain tended to select more bad decks relative to healthy controls, as seen from the learning curves, the statistical analysis identified neither group nor block as a significant factor that affected deck preference. This indicates no significant differences between the 2 groups, and both lacked evidence of significant learning across the 10 blocks during the task. These results are inconsistent with previous studies that identified significant group effects, in which patients won significantly less money and failed to adopt the advantageous decision-making strategy quickly learned by healthy controls [28,42,43]. A possible interpretation of this analysis is that the laboratory-in-the-field approach, which sacrifices experimenter control of the participants’ environment, causes greater variances in participants’ behavior, as revealed through their choices.

As expected, even with the relatively noisier data set, significant differences were identified in several cognitive components when comparing groups using the best-fitting models (VPP and ORL) measured by LOOIC in computational modeling analysis. The learning rate parameter in the VPP model determines how much weight is placed on past experiences of the chosen deck versus the most recent outcome from the deck. The chronic pain group demonstrated a much higher learning rate than the healthy controls, indicating that the recent outcome had a larger influence on the expectancy of the chosen deck and that forgetting was more rapid for individuals with chronic pain. The reward and punishment learning rates in the ORL model were used to update expectations after positive and negative outcomes, respectively. These 2 parameters account for the degree of the participants’ sensitivity to losses and gains.

Individuals with chronic pain demonstrated both elevated reward and punishment learning rates when fitting to the ORL model, suggesting that they gave more weight to recent outcomes, which is consistent with the results obtained from the VPP model. It was suggested in the study by Haines et al [37] that comparing the differences between the reward and punishment learning rates for the 2 groups was more useful, although they were defined separately. The larger the differences between the 2 learning rates, the more the learning is dominated by either rewards or punishments. The significantly higher reward learning rates that caused larger differences between the 2 learning rates in individuals with chronic pain suggested that they were more sensitive to gains over losses relative to healthy controls. In other words, individuals with chronic pain appeared to be more driven by rewards, which could be a possible reason that made them choose more cards from deck B (as seen in Figure 5), the bad deck with a higher reward magnitude but also a higher punishment magnitude. This finding differs from the result reported in the study by Elvemo et al [44], where people with chronic pain only demonstrated significantly reduced scores on reward responsiveness but not on the self-reported tendency to pursue rewards. It can be seen from Equation (12), that the outcome frequency weight and perseverance weight parameter in the ORL model collectively influence the total value of the expected value of each deck. Values for the outcome frequency <0 or >0 indicate that decision makers prefer decks with low or high win frequency, respectively. This value was >0 for the 2 groups and did not show significant differences, suggesting that both groups preferred decks with high win frequency (ie, decks B and D), which can be reflected in the learning curves plotted in Figure 5, where healthy individuals ended up selecting deck D the most and patients with chronic pain ended up selecting deck B the most. Values for the perseverance weight <0 or >0 indicate that decision makers prefer to switch or stay with their recently chosen decks. The mean value of this parameter for the healthy controls was >0, whereas this value for the patients with chronic pain was <0. Meanwhile, there was a significant difference between the 2 groups. This means that people with chronic pain were less persistent in their previous choices during the task. In this sense, our findings are in agreement with a previous study [33] where a simple heuristic model was proposed to discriminate between patients and healthy controls on IGT performance by tuning the degree of randomness and importance given to losses and gains. Patients with chronic pain in this study demonstrated significantly less persistent behavior, which was characterized by giving more emphasis to gains than to losses and increasing decision randomness. However, contrary to our expectation, decision makers with chronic pain presented lower decay rates, suggesting that they base decisions on longer histories than healthy controls. However, substantial existing studies have consistently reported impaired memory functions in patients with chronic pain [45,46], and memory complaints are one of the most common complaints in patients with chronic pain and cognitive deficits [47]. In addition, this result is inconsistent with the findings fitting to the VPP model, where decision makers with chronic pain had higher learning rates and, therefore, relatively worse memory. It was argued in the study by Ahn et al [26] that this situation where the parameters of a model with good model fit might not correctly reflect the underlying cognitive constructs could be caused by the insufficient number of trials such that insufficient information was extracted to reliably estimate the free parameters in the model; thus, it might be helpful to perform external tests in future research [48].

When analyzing the correlations between the cognitive parameters and self-reported pain experiences, we found that the learning rate in the VPP model was positively associated with the 4 pain measures, whereas the outcome sensitivity, response consistency, loss aversion, and weight parameter were negatively associated with the 4 pain measures. Higher reward learning rates in the ORL model could significantly predict higher self-reported pain severity and pain interference. Lower decay rates in the ORL model were only associated with higher self-reported pain interference, especially with the affective subdimension of interference. Given the correlations observed between the cognitive parameters and pain experiences, cognitive tasks might be an important tool to consider when evaluating individuals at risk of developing chronic pain conditions.

In summary, by recruiting participants on the web, administering the pain inventory and the IGT in natural environments over the web, and breaking down their performance into distinct psychological processes using computational modeling analysis, we revealed that participants with chronic pain displayed increased reward sensitivity and reduced choice persistency to their previous choices relative to healthy controls, and some of the cognitive parameters could predict the participants’ pain severity and pain interference. Compared with conventional statistical analysis of behavioral performance, computational modeling analysis revealed much more evidence of distinct differences in decision-making between individuals with chronic pain and healthy controls in the noisier laboratory-in-the-field environment.

### Limitations

First, only sex, age, and pain duration were collected in the demographic data, but no other measures of education or psychological status, such as anxiety and depression, were considered apart from pain severity and pain interference. It is known that experiencing chronic pain puts a person at increased risk of developing anxiety and depression disorders, and these 2 factors have been documented to have a significant influence on decision-making [49,50]. We did not consider the impact of medications on decision-making either (ie, we did have the participants’ medication information but did not exclude participants who were receiving medical treatments). Another important factor that could influence decision-making is environmental distractions, such as family members, distractions in the environment, and time of the day. It is also worth noting that virtual money may not be as effective as real financial incentives for the decision-making task. However, we did achieve good adherence to and compliance with the task. Furthermore, we recruited a mixed sample of participants who might have had various kinds of pain conditions that might cause various cognitive abnormalities and, thus, have different influences on decision-making. As a result, caution must be exercised when interpreting the results obtained and generalizing them to pain populations in specific environments and particular chronic pain conditions. Future research should conduct a more comprehensive assessment of the participants and analyze the potential impacts of the aforementioned variables. Finally, a sample size of 45 could be considered relatively small, especially given that our recruitment was not a laboratory-based paradigm. This may be the cause of the analyses that revealed results that were not consistent with the existing literature. We take this smaller scale of study as pilot research to validate our research methodology. The computational methods demonstrated a great advantage in distinguishing between the underlying cognitive processes of participants with chronic pain and healthy controls in that regard.

## Abbreviations

BPI-SF: Brief Pain Inventory–Short Form
HDI: highest–posterior-density interval
IGT: Iowa Gambling Task
LOOIC: leave-one-out information criterion
mPFC: medial prefrontal cortex
ORL: Outcome-Representation Learning
PVL: Prospect Valence Learning
REM: relations with others, enjoyment of life, and mood subdimension of the Brief Pain Inventory–Short Form
VPP: Values-Plus-Perseverance
WASW: walking, general activity, sleep, and work subdimension of the Brief Pain Inventory–Short Form
